# MET network in PubMed: a text-mined network visualization and curation system

**DOI:** 10.1093/database/baw090

**Published:** 2016-05-30

**Authors:** Hong-Jie Dai, Chu-Hsien Su, Po-Ting Lai, Ming-Siang Huang, Jitendra Jonnagaddala, Toni Rose Jue, Shruti Rao, Hui-Jou Chou, Marija Milacic, Onkar Singh, Shabbir Syed-Abdul, Wen-Lian Hsu

**Affiliations:** ^1^Department of Computer Science and Information Engineering, National Taitung University, Taiwan, R.O.C; ^2^Interdisciplinary Program of Green and Information Technology, National Taitung University, Taiwan R.O.C.; ^3^Institute of Information Science, Academia Sinica, Taiwan, R.O.C; ^4^Department of Computer Science, National Tsing-Hua University, Taiwan, R.O.C; ^5^School of Public Health and Community Medicine, University of New South Wales, New South Wales, Australia; ^6^Prince of Wales Clinical School, University of New South Wales, New South Wales, Australia; ^7^Innovation Center for Biomedical Informatics, Georgetown University, Washington, DC, USA; ^8^Department of Computer Science, Rutgers University-Camden, Camden, NJ, USA; ^9^Department of Informatics and Bio-Computing, Ontario Institute for Cancer Research, Toronto, Ontario, Canada; ^10^Graduate Institute of Biomedical Informatics, Taipei Medical University, Taiwan, R.O.C; ^11^International Center for Health Information Technology, Taipei Medical University, Taipei, Taiwan R.O.C.

## Abstract

Metastasis is the dissemination of a cancer/tumor from one organ to another, and it is the most dangerous stage during cancer progression, causing more than 90% of cancer deaths. Improving the understanding of the complicated cellular mechanisms underlying metastasis requires investigations of the signaling pathways. To this end, we developed a METastasis (MET) network visualization and curation tool to assist metastasis researchers retrieve network information of interest while browsing through the large volume of studies in PubMed. MET can recognize relations among genes, cancers, tissues and organs of metastasis mentioned in the literature through text-mining techniques, and then produce a visualization of all mined relations in a metastasis network. To facilitate the curation process, MET is developed as a browser extension that allows curators to review and edit concepts and relations related to metastasis directly in PubMed. PubMed users can also view the metastatic networks integrated from the large collection of research papers directly through MET. For the BioCreative 2015 interactive track (IAT), a curation task was proposed to curate metastatic networks among PubMed abstracts. Six curators participated in the proposed task and a post-IAT task, curating 963 unique metastatic relations from 174 PubMed abstracts using MET.

**Database URL:** http://btm.tmu.edu.tw/metastasisway

## Introduction

Metastasis refers to the spread of a cancer from its primary site to other parts of the body (secondary sites), while maintaining its malignant growth. It is often the major concern of patients and clinicians, as it results in the death of over 90% of cancer patients (1–3). However, prediction of metastasis is a highly challenging task due to the dynamic nature of cancers. Two tumors with the exact same diagnosis may differ in their progression, as one moves to a secondary site but the other does not. Recently, increasing awareness of biological signaling pathways and their role in metastasis has enabled life scientists to acquire a more comprehensive overview of the metastatic process. Studies have supported the potential use of gene-specific target therapies in treating metastasis. Additional clinical trials will be conducted to validate this finding by examining drug-treated patient samples.

Increased understanding of the roles of genes in the metastatic mechanism can lead to improved treatment of cancer patients through the control of metastasis. Nevertheless, the large volume of online studies and the complexity of obscure gene–cancer interactions stand as the major obstacle to improving insight into these relations. In light of this, we developed a METastasis network visualization and curation tool (MET) to assist metastatic researchers in accessing metastatic networks of interest while browsing through the large volume of studies in PubMed. With the use of MET, PubMed users can easily access the network information curated in our database that is related to the accessed abstracts. Users interested in contributing their knowledge and findings about the abstracts can register as volunteer curators on our website. Registered users can add metastasis-related concepts found in the articles, and the metastatic relations these concepts are involved in directly to our database.

Following our previous success in developing a text mining-based curation system as a browser extension to promote biomarker curation (4), MET is implemented as a Chrome browser extension to make it easy to install and update while keeping the entire curation process in PubMed. As of November 2015, a total of 963 relations among 174 PubMed abstracts have been curated in the database.

## MET features

**Figure 1.**
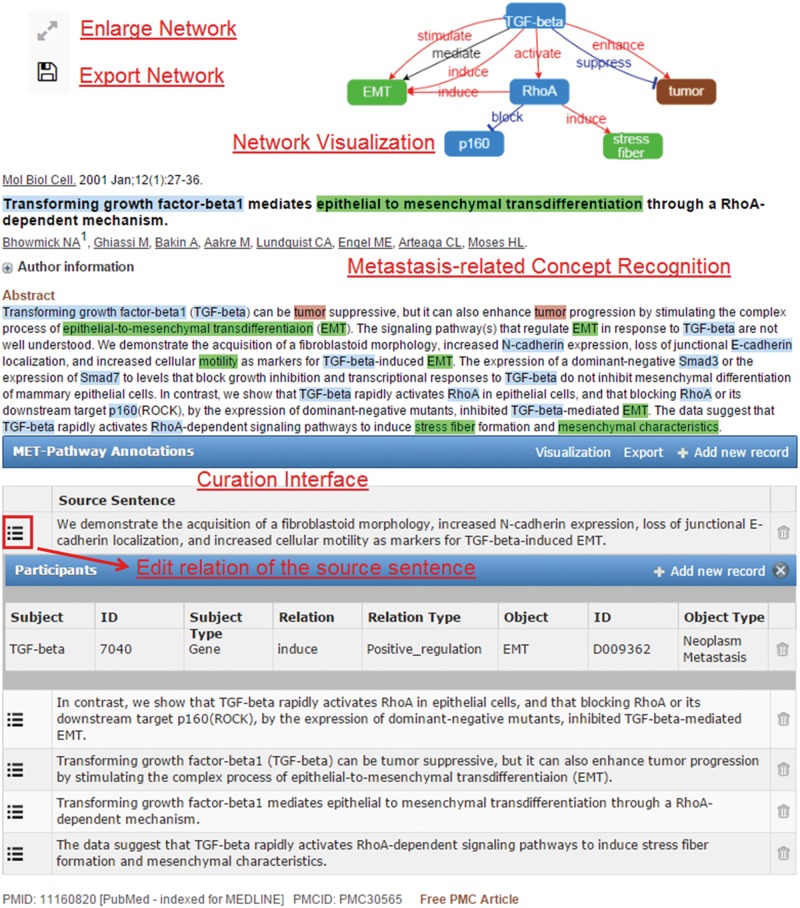
An article (PMID: 11160820) processed by MET.

The main features of MET include (i) display of the text-mined and user-curated recognition and normalization results of the metastasis-related concept terms described in an abstract, (ii) illustration of the extracted metastasis relation information as a network diagram and (ii) the curation interface for curators to update the recognition/normalization and relation extraction results. Figure 1 [TQ1] exhibits these features for an abstract (PMID: 11160820) on the PubMed web site. To generate the results shown in Figure 1, MET sends the article information to our text mining web services, which support the recognition of a wide range of biomedical concepts including gene, microRNAs, metastatic cancers, cytoskeletons, cell movements, cell adhesions, cancers, tissues and organs. Based on the recognized concepts, the relations among them are determined and sent back to MET for network visualization in the client’s browser.

**Figure 2.**
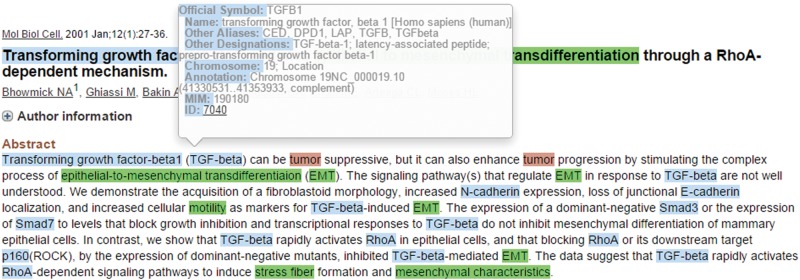
Summary information of the recognized TGF-beta gene.

When the mouse cursor hovers over a recognized concept, a brief pop-up summary of the concept will be displayed as shown in Figure 2. The summary information is based on the record in the Entrez Gene or the MeSH databases for the recognized gene/protein or cancers. The MET network visualization of a given abstract is constructed based on the information in the curation table below the abstract. Users can zoom in/out of the network or rearrange the network nodes using the cursor. The curated network information recorded in the curation table can be downloaded by clicking the ‘Export’ button.

If the user is registered as a curator, the curation function enables him/her to modify the extracted concepts and relations. Editable properties of a recognized concept include the concept type, and the normalized database ID for the concept.

**Figure 3.**
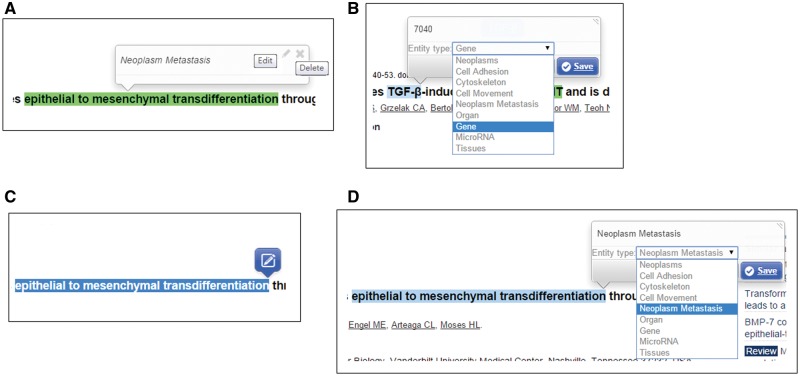
Curation interface for concept annotation.

Figure 3 presents the curation interface for the recognized concepts in MET. A curator can directly update the properties through the “Edit” button in the pop-up summary window (Figure 3A). In editing mode, the curator can change the concept type assigned either by our text mining system or other curators by selecting the correct one from the dropdown list (Figure 3B). He/she can also assign an Entrez Gene ID for the concept if it is a gene/protein/microRNA, or a MeSH term ID for a cancer concept. The ‘Delete’ button can be used to remove a recognized concept (Figure 3A). The curator can also add a new concept by first highlighting the words and then selecting its concept type through the curation interface (Figure 3C and 3D).

**Figure 4.**
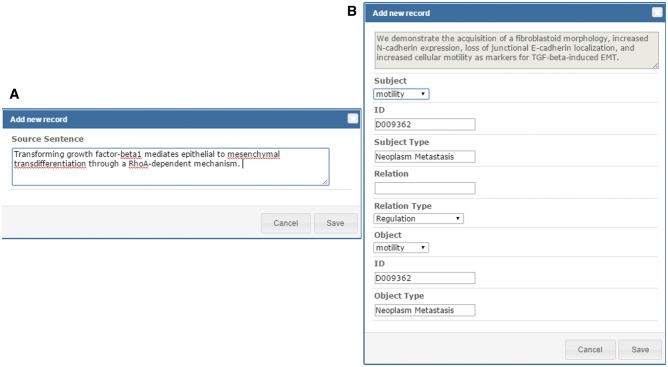
Curation interface for adding source sentences and relations.

As shown in Figure 1, the relations extracted by our text mining services are listed in the curation table below the abstract. The table includes all sentences that provide supporting evidence about the existing metastatic relations in the abstract. Curators can review the relation participants of the supporting sentence by clicking the ‘Edit’ button on the left side of the sentence. They can also add new source sentences and relations corresponding to a sentence that were not extracted by our services using the ‘Add new record’ button of the curation table as shown in Figure 1. To add a source sentence, the curator can either highlight the sentence in the abstract and click the ‘Add new record’ button to create a new record directly, or use the ‘Add new record’ interface shown in Figure 4A and enter the text of the sentence. Figure 4B shows the curation interface for a relation involving two concepts described in a curated sentence. A curator can select the participants in the dropdown list and set their relation type. Once curators confirm and save the curation results, the results are submitted and stored in our database. The curated binary relations will be integrate into biological events and illustrated in the MET network.

## Integrated networks of multiple abstracts

MET is capable of integrating individual networks derived from several abstracts. To view the integrated network, a user can submit the PMID of interest to the PubMed database, and change the display format from the default ‘Summary’ format to the ‘Abstract’ format as shown in Figure 5A. The integrated MET network can be shown in a full-size window as in Figure 5B by clicking the ‘Enlarge Network’ button. The user can zoom in/out or rearrange the nodes in the integrated network using the mouse.

**Figure 5.**
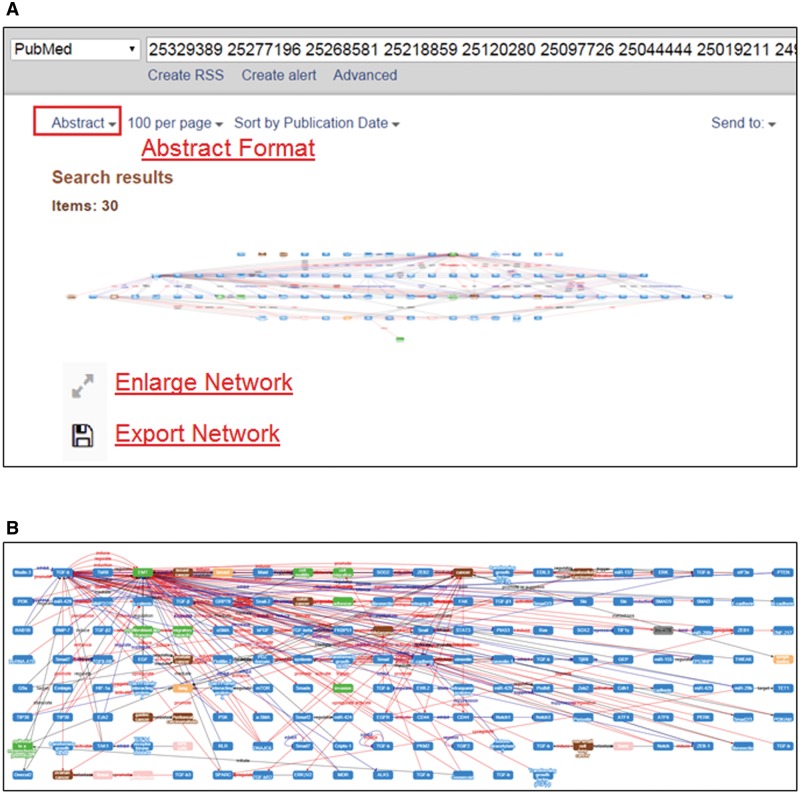
Integrated pathways of multiple abstracts by MET.

## Curation tasks of MET

### Target curation concept types

**Figure 6.**
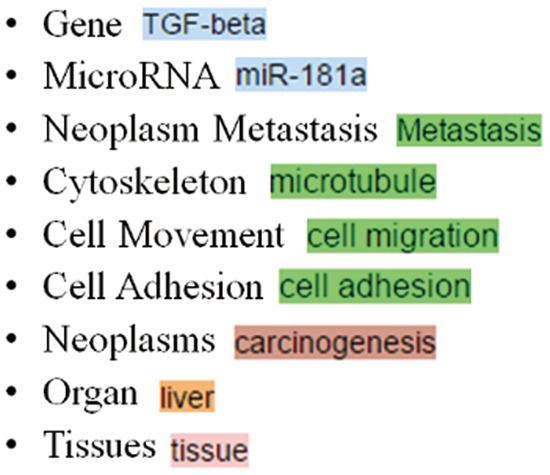
The colors used for different concept types in MET.

Table 1 summarizes the nine types of biomedical concepts a MET curator should curate. As shown in Figure 6, MET uses different colors to depict the concepts recognized by our services and curated by curators.

**Table 1.** Concept description and instance

Concept Type	Description	Example
Gene	The gene, gene product and microRNA names	TGF-β
MicroRNA		miR-181a
Neoplasm Metastasis	Metastasis is a complex disease containing series of biological processes. Therefore, descriptions related to cytoskeleton, cell movement and cell adhesion are also considered as instances of metastasis.	Metastasis
Cytoskeleton		Stress fiber
Cell Movement		Cell aggregation
Cell Adhesion		Cell adhesion
Neoplasms	The cancer names	Liver cancer
Organ	The organ names	Liver
Tissues	The tissue names	Adipose tissue

### Target curation event types

The curatable events for constructing a MET network are summarized as follows.

- Gene → Gene → Neoplasm Metastasis: The positive or negative regulation between genes (including microRNAs) resulted in the regulation of metastatic cancers (including concepts of cytoskeleton, cell movement and cell adhesion).
- Gene → Gene → Neoplasms ⇒ (Tissues | Organs): The positive/negative regulation or regulation between genes (including microRNAs) caused a cancer to metastasize to a certain organ or tissue. The relation (Neoplasms ⇒ Tissues | Organs) indicates that the relation involved a cancer concept as its subject, a tissue or organ concept as its object and a metastasis concept as its trigger.

As not all articles contain all the information required, the following relations, deconstructed from the above events, are provided to curators as suggestions to be used when they perform event curation using MET.

- (Gene | MicroRNA) → (Gene | MicroRNA)
- (Gene | MicroRNA) →(Neoplasm Metastasis | Cytoskeleton | Cell Movement | Cell Adhesion)
- (Gene | MicroRNA) → (Neoplasms)
- (Neoplasms) ⇒ (Tissues | Organ)

The symbol ‘⇒’ indicates a metastasis relation, and the symbol ‘→’ means a positive/negative regulation, or neutral regulation if the mode of the control cannot be determined from the context.

### Curation tasks for the BioCreative V interactive track

The BioCreative Interactive task (IAT) is a track designed to engage text-mining curation systems with potential curators by providing a communication channel between the bio-curation and the text mining communities (5). As one of the participants of the IAT of BioCreative V, we recruited 17 MET curators from different countries with the help of the user advisory group of IAT. Six of the curators (listed as the sixth to the 11th authors) joined and completed the proposed curation tasks. Prior to the IAT, curators were trained with annotation guideline and tutorials to familiarize them with MET and brat usage, along with the suggested procedures for the curation tasks (e.g. completing the concept curation first before stepping into the relation curation).

To collect articles for our curation tasks, the query term ‘EMT [title/abstract] AND TGF-β [title/abstract]’ was used to search PubMed for abstracts related to metastasis biological processes. This query retrieved 949 abstracts, 300 of which were randomly selected as the curation dataset for IAT. The dataset was split into six equal sets, with one set assigned to each curator as shown in Table 2.

**Table 2.** The curation dataset assigned to each curator

Curator #	MET Week1	Manual Week1	MET Week2	Manual Week2
C1	G1	G3	G2	G4
C2	G2	G4	G1	G3
C3	G3	G5	G4	G6
C4	G4	G6	G3	G5
C5	G5	G1	G6	G2
C6	G6	G2	G5	G1

Following the IAT process, the curation tasks were divided into two phases over 2 weeks. During each phase, the curators were asked to complete the following two tasks.

#### MET-assisted curation task

The curators used the MET-enabled browser to curate articles directly on the PubMed website through the MET text mining services. MET highlights all recognized concepts and visualizes the constructed networks in PubMed as illustrated in Figure 1. The curators were asked to verify all recognized concepts and relations, and add any missing concepts or relations if necessary.

#### Brat-based manual curation task

The curators used the brat annotation tool (6) to manually complete the assignment. The abstracts assigned to each curator were uploaded in advance on our brat website. The abstracts were not pre-processed by our text mining services to recognize concepts and relations, and the curator was asked to first manually annotate all existing concepts followed by the relations among them. During the first week, curators were asked to annotate the original abstracts without any aid. In the second week, curators worked on a second batch of articles, which had already been annotated by another curator, but without knowing those annotations were manually created.

## Results and discussion

### IAT curation results and observations

Table 3 lists the number of curated abstracts for the six sets after the 2 weeks. Five curators successfully completed the curation task, while one had to drop out before the end (indicated as ‘n/a’ in Table 3). MET-assisted and brat-based curation respectively averaged 8.4 and 9.5 abstracts per hour.

**Table 3.** The number of completed abstracts for each dataset

Group #	MET Week1	MET Week 2	Manual Week 1	Manual Week 2
G1	6	7	10	15
G2	4	6	4	7
G3	10	n/a	8	8
G4	n/a	13	8	6
G5	10	13	16	n/a
G6	10	5	n/a	13
Total	40	44	46	49

A direct comparison of efficiency between MET and brat tool may not be completely appropriate. Brat is a mature tool with an easy-to-use annotation interface, and some of our curators already had experience using the tool for annotation. In contrast, MET is under development and many new features were implemented and integrated during the IAT. As shown in Table 3, surprisingly the number of abstracts completed by brat-based manual curation was higher than that completed by MET-assisted curation in both sessions. Post-task discussions with the curators and a review of the curation results resulted in the following observations.

First, we found that in the brat-based manual curation task, most curators only annotated the locations of the concepts, but did not normalize the annotated concepts to proper database IDs. Although the guidelines do ask them to annotate the IDs, some curators failed to do so. The brat interface does not support the display of the detailed information associated with the assigned ID could be one of the possible reason that the curators overlook the normalization task. In the scenario, if the curator does not familiar with the information associated with that ID, he has to inquire the related information from Entrez Gene by himself to check the correctness. Second, MET provides a comprehensive information set, including all of the metastasis-related concepts recognized by our text mining services, their normalized IDs and the relations among them. Curators had to go through all of these data and correct them if necessary. In contrast, in the manual curation task, curators could focus on annotating relations and the concepts involved within the same sentence, while ignoring all other concepts that exist in the same abstract.

In addition, we observed that without any text-mining assistance, curators might generate inconsistent annotation of concepts and relations even when given access to the annotation guideline. For example, we observed that one curator annotated ‘more’ as a trigger word in sentences like ‘… metachronous metastasis was observed **more** frequently in the cases with high PRL-3 expression’. In our definition, a trigger word should be a verb or a nominal verb that causes direct relations between the concepts. Therefore, in this case, the curator should not annotate the adverb word ‘more’. One of the most crucial issues is that curators might forget to annotate essential concepts and relations in their manual curation task. For instance, they might forget to create the connection between the relation trigger and entities to construct a relation in brat. An automatic qualification test system, as introduced by (7), for assessing the candidate curator’s comprehension of the annotation guidelines based on real examples from our IAT may be required before they are allowed to access the curation interface.

Another interesting issue is that comparing the manual curation results for both weeks shows no significant increase in the number of completed articles. In addition, providing previous concept annotations do not reduce the effort required on the part of the curators, because the curators tend to check all annotations by themselves. The curation task is designed to have curators provide extensive annotations of all mentions. Nevertheless, curators would only be interested in annotating the subset that is most relevant for curation in practice. This observation supports the argument of the IAT organizers (5) that there is an important difference in the way a human curator and the text mining system approaches the curation task. We believe that, although concept annotation is rather important for text miners, it might not be as essential for experienced curators. Therefore, concealing relatively less informative data for curators would be preferable in designing the curation interface. However, this might result in the loss of important information, thus a comprehensive design is required.

Finally, to evaluate inter-annotator agreement, the datasets assigned to different curators were overlapped as shown in Table 2 to give different curators the chance to annotate the same article sets in both the MET-assisted and brat-based manual curation tasks. However, because articles are displayed in different orders in PubMed (by publication date) and the brat system (by file name), no overlapping articles were found when we tried to evaluate the inter-annotator agreement using article sets in the MET-assisted and manually curated results. Therefore, the results from the two curation methods cannot be directly compared.

The manually curated data set can be downloaded from the BioC web site (http://bioc.sourceforge.net/) or the project page of MET https://sites.google.com/site/hjdairesearch/Projects/met.

### Post-IAT curation results and observations

Within the 2 weeks of the IAT, the curators reported some problems with MET and our text mining services, including the boundary errors of concept annotations and the inconsistency between the curated relations and the visualized networks. These errors caused some delays in the curation process but valuable curator feedback also helped us to improve MET. For example, some concepts annotated by the curators were not available in the dictionaries used for concept recognition, which gave us the chance to improve the recall of our dictionaries. Before the start of the second week, we made a major update to MET to fix several bugs reported in the first week. This enhancement is reflected by the increased number of curated abstract in the second week as illustrated in Table 3. It also motivated us to redesign the curation task and the curation interface of MET for the post-IAT task.

In the post-IAT task, the definition of the MET-assisted curation task is updated as follows.

#### MET-assisted post-IAT curation task

The curators used the MET-enabled browser to conduct their curation directly on the PubMed website with the assistance of our text mining services. MET highlights all recognized concepts and visualize the constructed networks. The curators were asked to verify all recognized concepts/relations and add any missing concepts if necessary. When adding a missing relation, the curators were asked to first curate the supporting sentence, and then curate the relation itself.

There is one significant difference in the post-IAT task–an additional curation step to first curate the supporting sentence, followed by the curation of the relation itself. The same six curators were invited to join the post-IAT task, and five of them completed the assignment. They were asked to complete as much of the remainder of the two sets assigned as possible in two hours. Using the new curation model, five curators finished their curations and generated 702 relations from 116 unique abstracts, which is almost twice the number of abstracts curated in the IAT MET-assisted curation task (64 abstracts and 298 relations). The curation rate for the post-IAT task was 12.3 abstracts/hour, which is even higher than the curation rate reported in the IAT brat-based manual curation task (9.5 abstracts/hour). We believe this improvement can be attributed to the use of the new curation model in the post-IAT task.

In the original curation model used in the IAT MET-assisted curation task, the curator selects participants from a dropdown list including all recognized concepts mentioned in the abstract, while simultaneously provide the supporting sentence. Using the new model, however, the curator curates the supporting sentence first, and then curates the relation participants. Although the new curation model seems to be more time-consuming, the results demonstrate the positive impact of the additional curation step. One of the main reasons that the new model can boost the curation rate is that, by first identifying the sentence, the curator can then select the participants from a dropdown list that contains many fewer candidates. This is also the reason that some of the relation extraction tasks in text mining, such as protein–protein interaction extraction, first define a subtask to identify the target sentence (8). As described in the previous section, the new curation model also led to a change in the design of the MET curation interface. The new model first displays the source sentence, followed by its recognized relations, while the old model directly shows all of the relations in the curation interface.

## Text mining services

### Concept recognition

For the recognition of gene mentions, our text mining services integrated the results from PubTator (9) and our multistage gene normalization system (10, 11). For the recognition of microRNA, a pattern-based approach is employed (12), which achieved an *F*-score of 0.97 on the miRNA-Test-Corpus (13). For the rest of the concept types, dictionary-based recognizers were implemented along with the corresponding terms collected from MeSH and the terms manually added by our in-lab biologists. The terms curated by our curators were also manually examined for inclusion into their corresponding dictionaries.

### Relation extraction

A MET network consists of different biological relationships between the concepts such as gene-gene regulation, gene-cancer regulation and the organs of the metastasis. Our text mining service uses the principle-pattern-based approach (14, 15) to extract these relations. One of the main reasons we employ the pattern-based method is the lack of a metastasis-gene relation corpus. The relational patterns were semi-automatically generated using a random walk algorithm and manually verified by our in-lab biologists. The generated patterns consist of the concept type tags such as ‘<GENE>’ and ‘<METASTASIS>’ and the relation predicates such as ‘inhibit’ and ‘regulate’. For example, the pattern ‘<GENE> <POSITIVE_REG>  <GENE>’ can be used to extract positive regulation relations. To evaluate the performance of our principle-pattern-based relation extraction approach, we assume that the boundaries of concepts are given. The 64 abstracts curated by our curators in IAT were used as the testing set, and the relation extraction component achieved the precision/recall/F-measure of 0.68/0.77/0.72.

## Conclusion

We have developed MET, a network visualization and curation system that can facilitate the curation of metastatic networks. Critical feedback was received from MET users through the participation of the BioCreative IAT. Overall, users found MET to be a unique and easy-to-use tool for metastatic network curation. Participation survey results indicated most of the curators thought that the annotated concepts and visualized networks provided by MET were extremely helpful in suggesting which concept was involved in the metastasis network. MET can help them efficiently extract the metastasis knowledge from the abstracts. The concept types provided by MET are also considered to be unique, because no other tools provide so many concept types to assist in the curation of studies for network information. We believe that with further enhancement, MET could prove very useful, especially in the construction of a database for metastasis networks. MET also has great potential for application to the curation of other concepts.

Future work will focus on correcting the inconsistent annotation boundaries of concepts. Furthermore, based on the responses from the MET curators in IAT, we plan to improve MET to recognize additional biological concepts such as drug names and other diseases apart from cancers, and the relations among these concepts such as disease–cancer and drug–cancer relations, aiming to provide a more comprehensive view of the MET networks. All recognized relations would be clustered based on a control vocabulary such as the GENIA Event Ontology (16) for generalizing the integrated networks to improve ease of comprehension. We also intend to implement an automatic test system for the assessment of curator qualifications for the MET curation task, and integrate the result section recognizer (17) in our text mining service to focus on sentences in the ‘Results and Conclusion’ section of an abstract, thus mitigating the risk of extracting possibly misleading relations from the ‘Background or Method’ sections of the abstract.
